# Isolated pseudo‑abducens palsy and contralateral occipital headache with thalamic stroke: A case report and mini‑review of the literature

**DOI:** 10.3892/mi.2024.142

**Published:** 2024-02-22

**Authors:** Jamir Pitton Rissardo, Hossam Tharwat Ali, Asad Riaz, Ana Leticia Fornari Caprara

**Affiliations:** 1 Department of Neurology, Cooper University Hospital, Camden, NJ 08103, USA; 2 Qena Faculty of Medicine, South Valley University, Qena, Qena Governorate 83621, Egypt; 3 Ayub Teaching Hospital, Islamabad, Islamabad Capital Territory 44000, Pakistan; 4 Department of Medicine, Federal University of Santa Maria, Santa Maria, RS 97105-900, Brazil

**Keywords:** pseudo-abducens palsy, sixth nerve palsy, abducens palsy, thalamic stroke, thalamic pain syndrome, stroke

## Abstract

The abducens nerve (sixth cranial nerve) is essential for lateral eye movement, and its malfunction can cause a variety of issues with vision. Pseudo-abducens palsy is a rare neurological condition that causes a limitation in eye abduction, while the abducens nerve is still functioning. Thalamic pain syndrome, a severe complication of cerebrovascular events, presents as intense neuropathic pain provoked by temperature fluctuations. Although thalamic strokes are infrequently associated with ocular abnormalities, some studies suggest an association between isolated pseudo-abducens palsy and thalamic infarctions. The present study describes the case of a 38-year-old male patient with 1-day progressive diplopia and occipital headache who had abducens palsy on the left side as a result of a right thalamic infarction. The patient had a 10-year history of smoking and a 1-year history of hypertension, which was poorly controlled. The diagnosis was supported by a neurological examination, imaging and stroke etiology investigations. The patient recovered well within 5 days, highlighting the good prognosis of an acute thalamic presentation. In addition, a mini-review of the literature was performed and two similar reports were identified upon searching the literature using the Embase, Google Scholar, Lilacs, Medline, SciELO and ScienceDirect databases. On the whole, the present study demonstrates that understanding the complex neuronal connections inside the thalamus is critical for a proper diagnosis and appropriate intervention strategies in patients with thalamic stroke with oculomotor impairments. Further research is required to elucidate the underlying causes and develop treatment techniques for thalamic infarction consequences.

## Introduction

Cerebrovascular accidents (CVAs) constitute a major cause of morbidity and mortality worldwide. There are >700,000 cases of CVAs in the United States annually (1). The thalamus, which is involved in numerous brain functions, including memory, emotions, the processing of sensory inputs and sensorimotor functions, can be involved in CVAs, either alone, or with infarcts in other brain structures (2,3). Thalamic pain syndrome is an adverse occurrence following of a CVA that is concentrated neuropathic pain triggered by temperature fluctuations. Patients frequently experience hyperalgesia and allodynia. Following a CVA, the onset of a patient's symptoms is frequently delayed. A patient with a thalamic CVA may not experience substantial discomfort for months or years following the stroke. The thalamic pain syndrome is a form of central post-stroke pain in all situations (1).

As reported in the study by Cambier (4), Dejerine and Roussy first described the classic thalamic syndrome in 1906. Following their description, a number of modifications and other clinical manifestations related to the thalamus were included. In this manner, ocular movement abnormalities, which are uncommonly associated with thalamic strokes, were already associated with the impairment of vertical gaze with or without affecting the sixth cranial nerve (5). The abducens nerve (sixth cranial nerve) innervates the ipsilateral lateral rectus muscle and is thus involved in the abduction or lateral movement of the eye. Abduction palsy causes double vision and horizontal binocularity, and worsens when looking to the same side of the lesion (6). Pseudo-abducens palsy is a neurological limitation of abduction when the abducens nerve is intact. This rare condition may be observed when spontaneous eye movements exhibit an impaired lateral gaze, although the vestibulo-ocular reflex (VOR) exhibits complete abduction (7).

Isolated pseudo-abducens palsy secondary to thalamic stroke was rarely reported in the literature. Only two case reports of the individuals with infarction of the thalamus presenting with isolated sixth cranial nerve palsy have been described to date, at least to the best of our knowledge (8,9). The present study describes the case of a middle-aged male patient who presented with 1-day progressive diplopia and occipital headache who had abducens palsy on the left side as a result of a right thalamic infarction.

## Case report

A 38-year-old right-handed male presenting with diplopia was admitted to University Hospital of Santa Maria (Santa Maria, Brazil). The individual reported that this clinical manifestation began within 1 day and progressively worsened. He also complained of a new-onset mild occipital headache on the right side unrelated to eye movements or photo- and phonophobia. He was a construction worker, and there was no history of neurological diseases in his family. The patient had a history of smoking for years and 1 year of hypertension, for which the patient was not compliant with the medications.

The subject was fully conscious, and oriented to time and place. The neurological examination revealed left abducens palsy with intact bilateral vertical and right horizontal to voluntary movements. In addition, the diplopia was more prominent when the patient looked toward the left side. Monocular horizontal ductions exhibited the same abduction limitation as when viewing with both eyes. The oculocephalic reflex was normal. Palpebral oculogyric reflex was normal. Myosis, ptosis, nystagmus and skew deviation were not present. Muscle strength was normal, graded as five [Medical Research Council Muscle Power Scale (10)] all over the four extremities. Deep tendon reflexes were normal. A sensory examination yielded normal results and the rest of the cranial nerves were intact. There were no cerebellar signs. The remaining physical examination was normal.

**Figure 1:**
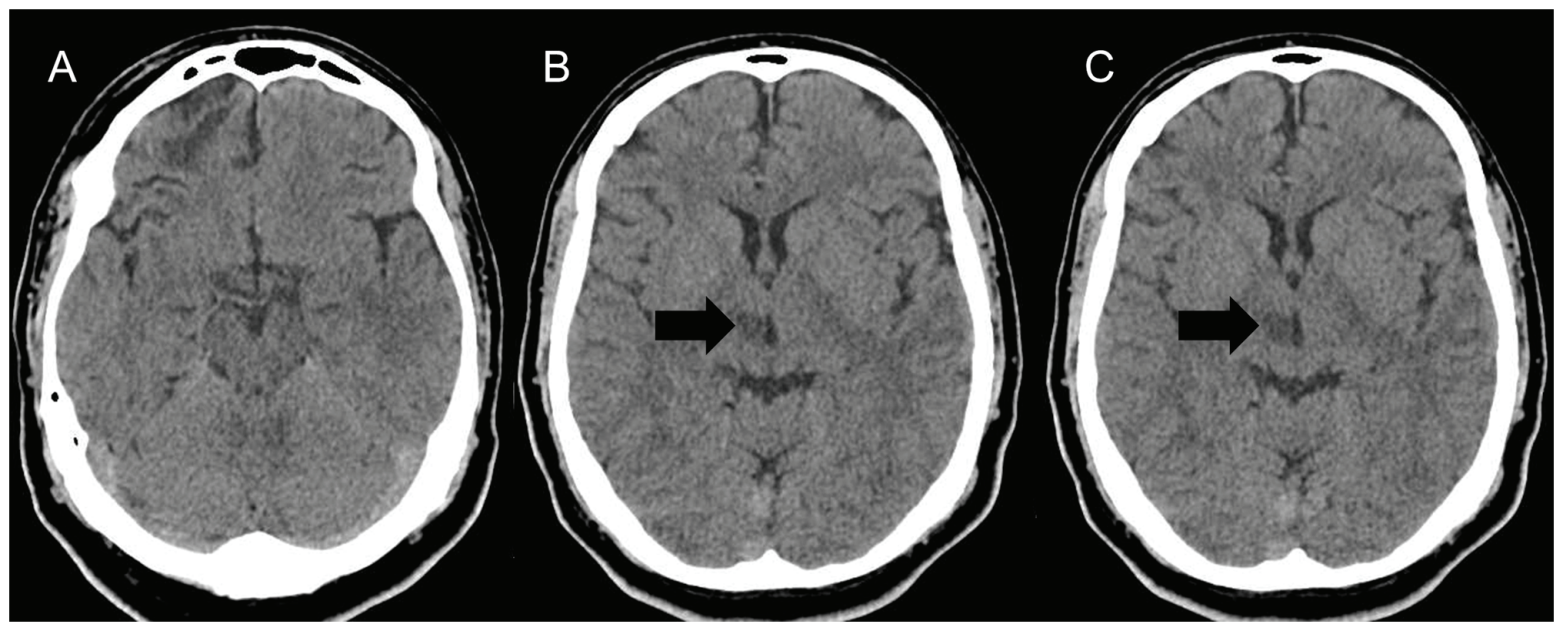
Neuroimaging illustrating thalamic infarction (arrow). (A) Level of the lower midbrain, (B) level of the upper midbrain, and (C) level of the lower basal ganglia images of an axial cranial computed tomography scan.

Laboratory tests, including a complete blood count, platelet count, prothrombin time, partial thromboplastin time, serum lipids and inflammatory test results were within normal limits; an investigation for hypercoagulable disorders yielded negative results. A cranial computed tomography (CT) scan revealed a right thalamic infarction (Fig. 1). A brain magnetic resonance imaging (MRI) demonstrated a lesion of 1.2x1 cm in size (data not shown; image not available), limited to the thalamus, which was hyperintense on T2-weighted and fluid-attenuated inversion recovery, consistent with acute ischemic infarct without affecting the subthalamic or midbrain area. Cerebrospinal fluid analysis and culture yielded normal findings.

Upon a stroke etiology investigation, cardiac monitoring throughout his admission was normal. A transthoracic echocardiography revealed mild left ventricle hypertrophy with a normal ejection fraction (56%), left segmental contraction and valves. A CT angiography of the head and neck yielded normal findings. The patient was managed in the stroke unit of the hospital and on day 5 of admission, the patient fully recovered his eye movements and was discharged.

## Discussion

The thalamus is a diencephalic grey matter structure whose circuits represent a gateway for input and output, in which nuclei integrate sensory, motor and behavioral signals of the cerebral cortex with other pathways (4). This complex structure is supplied by four arteries, three derived from the vertebrobasilar system (paramedian thalamic-subthalamic, thalamogeniculate and posterior choroidal arteries) and one derived from the posterior communicating artery (polar artery) (3). Therefore, thalamic lesions can present as a number of syndromes depending on the compromised vascular territory.

Infarctions of the thalamus correspond to ~3% of all ischemic stroke cases (3). Notably, thalamic strokes constitute 20-33% of post-stroke centralized pain cases (1). Moreover, when the stroke is specifically analyzed in the diencephalon, abnormalities in vertical or horizontal eye movements following ischemia in this region are only observed in 5% individuals. In this manner, ocular abnormalities are rarely reported in thalamic strokes (5).

In 1959, Fisher (11) were the first to characterize the term ‘sixth nerve pseudopalsy’, which was described as a paralysis of the voluntary ocular abduction that could be overcome by ice water caloric stimulation. Following that study, numerous reports with pure thalamic lesions have demonstrated oculomotor defects associated with thalamic infarctions. These ocular manifestations include the ocular tilt reaction, skew deviation, supranuclear third cranial nerve palsy and upward gaze palsy (5,8). Furthermore, experimental studies with provoked lesions in the thalamus of models support the hypothesis that the thalamus may be involved in oculomotor control (12).

To date, at least to the best of our knowledge, only a few cases of patients with thalamic infarctions who developed isolated pseudo-abducens palsy have been reported in the literature. In the present study, after performing a thorough review of the literature published in the English language, two cases were identified and these were compared with the present case (Table I) (8,9). A literature search was performed in Embase, Google Scholar, Lilacs, Medline, Scielo and ScienceDirect, using a set of terms that included the thalamus, thalamic stroke, stroke, and abducens palsy.

**Figure 2:**
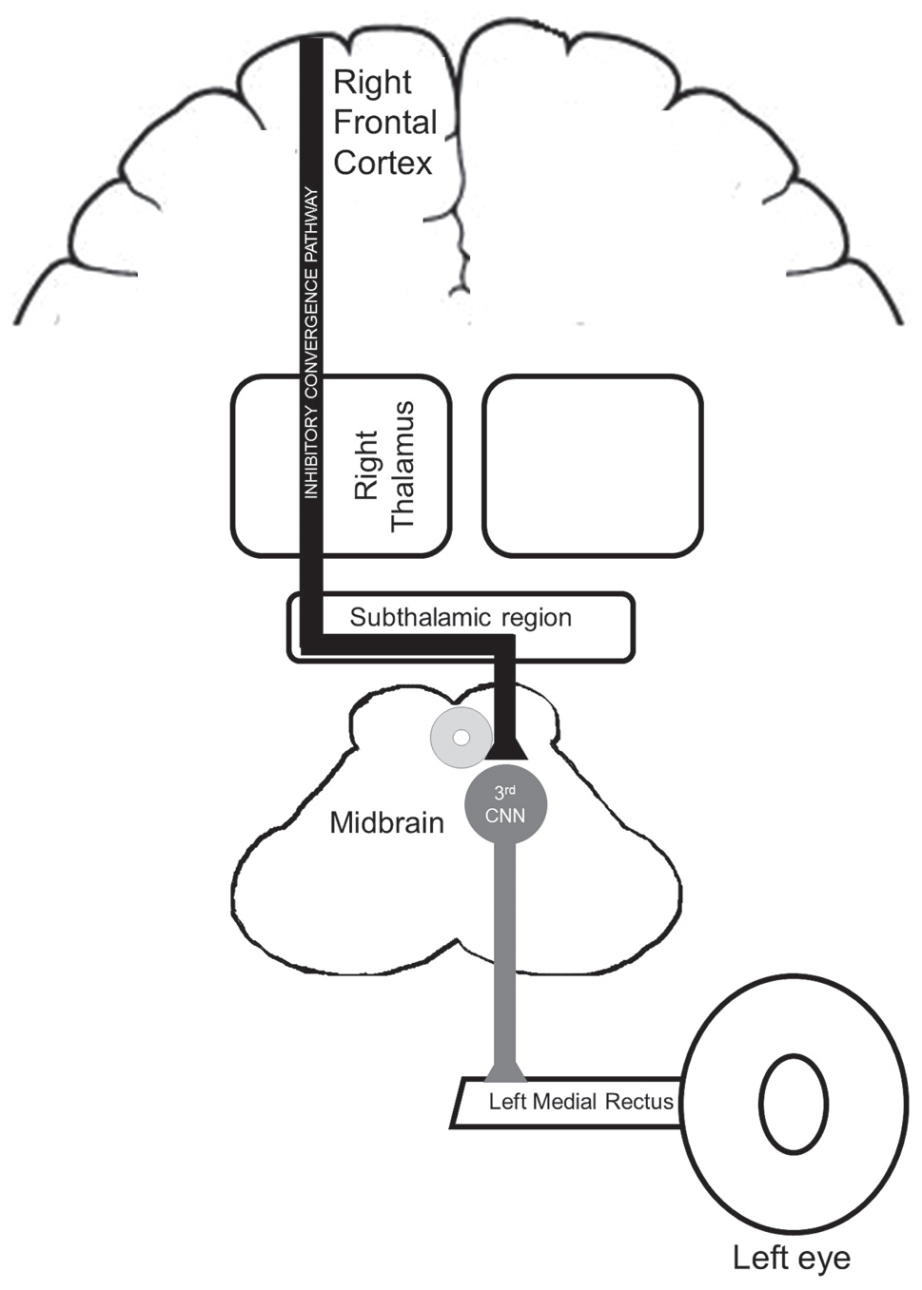
Schematic diagram illustrating the possible mechanisms of the isolated thalamic infarction causing pseudo-abducens palsy. First, the inhibitory convergence pathway begins in the frontal and supplementary eye field in the motor cortex. Second, the pathway passes through the thalamus and decussates in the subthalamic region. Third, the inhibitory convergence pathway possibly controls the third cranial nerve nucleus localized in the midbrain. 3rd CNN, third cranial nerve nucleus.

In the cases presented in Table I, the majority of patients exhibited some notable characteristics. First, their presentation included horizontal diplopia and contralateral headache. Headaches are common in individuals with stroke, and are more common when the infarction is in the thalamus (13). Secondly, the ocular finding contralateral to the stroke supports the hypothesis presented in the study by Wiest *et al* (9), who proposed that the oculomotor features could occur secondary to an interruption of the inhibitory convergence pathway that goes through the paramedian thalamus and probably decussates in the subthalamic region (Fig. 2) (8,9). Another key finding was the good prognosis of this acute thalamic presentation from the time from admission to full recovery only required a few days.

The paramedian thalamic-subthalamic artery is the second most common vascular territory of the thalamus to be affected by stroke, which is associated with pure motor abnormalities along with third cranial nerve involvement and vertical gaze abnormalities (3). In this manner, the nuclei in this vascular territory are probably connected with the frontal and supplementary eye field in the motor cortex (12). Thus, as previously demonstrated, individuals with an isolated stroke of the thalamus who present with oculomotor deficits probably have lesions in the intralaminar and dorsomedial nuclei (8,12).

Vasculopathy and non-vasculopathy risk factors exist for abducens nerve palsy in adults. Diabetes and other medical disorders are among the top prevalent vasculopathy risk factors in elderly adults. However, non-vasculopathy reasons, which can affect both adults and children, may include a variety of elements such as trauma, inflammation, or compression (5).

The onset of pseudo-abducens palsy is markedly influenced by lesions in the vicinity of the midbrain-diencephalic junction. Both convergence-retraction nystagmus and pseudo-abducens palsy are most likely signs of aberrant vergence activation. The thalamus may be the passageway for inhibitory descending processes that terminate in the subthalamic area (14). It is noteworthy that horizontal diplopia resulting from an isolated abducens (sixth) nerve palsy can also manifest as the first sign of multiple sclerosis; thus, this should also be kept as a differential diagnosis (15). Numerous conditions can present similarly to the thalamic pain syndrome including chronic pain syndrome, idiopathic peripheral neuropathy, multiple sclerosis, brain space-occupying lesions and lateral medullary infarction. However, thorough history and physical examination are essential to deem these diagnoses less likely (1,2).

In conclusion, the present study describes the case of a middle-aged male patient with unilateral pseudo-abducens palsy and contralateral headache as manifestations of acute thalamic stroke. The present case report suggests that thalamic infarctions should be listed as a probable cause of pseudo-abducens palsy. Moreover, patients presenting with acute thalamic syndrome probably have a good prognosis. The case study highlights the need for an in-depth understanding of the complex neural pathways within the thalamus, as well as the various clinical outcomes that can occur following a thalamus infarction. Further investigations are necessary in order to better understand the underlying mechanisms and to optimize treatment approaches for patients presenting with similar complications associated with thalamic strokes.

## Figures and Tables

**Table I:** Case reports of individuals who developed isolated pseudo-abducens palsy secondary to a thalamic stroke.

	Study		
Parameter	Wiest *et al* (9)	Ghasemi *et al* (8)	Present study
Age, years/sex	57/M	31/M	38/M
Initial clinical symptoms	Coma, pneumonia	12 h of horizontal diplopia associated with unsteady and contralateral occipital headache	24 h of horizontal diplopia with contralateral occipital headache
Comorbidities, medications in use, and risk factors for cerebrovascular diseases	None	Smoking	Smoking, hypertension
Key findings in the neurological examination	Bilateral abducens palsy	Right abducens palsy	Left abducens palsy
Neuroimaging requested	MRI	MRI	CT scan, MRI
Lesion site	Bilateral thalamus	Left thalamus	Right thalamus
MRA and CTA	NR	Normal	Normal
Stroke etiology	NR	Embolic stroke of undetermined source	Lacunar infarct probably secondary to hypertension
Time since clinical presentation and recovery (days)	NR	Three	Five

CT, computed tomography scan; M, male; MRI, magnetic resonance imaging; NR, not reported; MRA, magnetic resonance angiography; CTA, computed tomography angiography.

## Data Availability

The datasets used and/or analyzed during the current study are available from the corresponding author on reasonable request.
